# Supplementary material for: Deletion of wfs1 Impairs Oligodendrocyte Precursor Cells Dorsal Distribution and Myelination Through the wfs1-hmgcs1 Axis in Zebrafish
Source: Biology (Basel). 2026 Aug 16;15(16):1402. doi: 10.3390/biology15161402 (PMC13510151; doi:10.3390/biology15161402)
Supplement: Supplementary file 1 [file biology-15-01402-s001.zip › File S1-Transcriptome sequencing data and differentially expressed genes (DEGs) in wfs1-- zebrafish at 5 dpf/2.KEGG/wfs1KOvsWT/GSEA/WT/ENDOCYTOSIS/ENDOCYTOSIS(DRE04144).html]

Details for gene set ENDOCYTOSIS(DRE04144)[GSEA]

|  || Dataset | deg.sample |
| Phenotype | sample.cls |
| Upregulated in class | WT |
| GeneSet | ENDOCYTOSIS(DRE04144) |
| Enrichment Score (ES) | -0.24620187 |
| Normalized Enrichment Score (NES) | -1.4943845 |
| Nominal p-value | 0.0 |
| FDR q-value | 0.8692797 |
| FWER p-Value | 0.722 |
Table: GSEA Results Summary

  

Fig 1: Enrichment plot: ENDOCYTOSIS(DRE04144)      
 Profile of the Running ES Score & Positions of GeneSet Members on the Rank Ordered List

  

| PROBE | DESCRIPTION (from dataset) | GENE SYMBOL | GENE\_TITLE | RANK IN GENE LIST | RANK METRIC SCORE | RUNNING ES | CORE ENRICHMENT || 1 | 406837\_1 | rnf41 |  |  | 120 | 4.652 | 0.0147 | No |
| 2 | 336368 | vps37a |  |  | 138 | 4.569 | 0.0323 | No |
| 3 | 792229\_1 | sh3glb1a |  |  | 466 | 3.092 | 0.0343 | No |
| 4 | 792828\_1 | pard6a |  |  | 627 | 2.729 | 0.0400 | No |
| 5 | 556226\_1 | cdc42l2 |  |  | 680 | 2.623 | 0.0488 | No |
| 6 | 336426 | chmp1b |  |  | 758 | 2.459 | 0.0562 | No |
| 7 | 793158\_1 | cdc42l |  |  | 826 | 2.351 | 0.0634 | No |
| 8 | 406684\_1 | vps26b |  |  | 879 | 2.266 | 0.0708 | No |
| 9 | 573376 | hspa8 |  |  | 894 | 2.238 | 0.0792 | No |
| 10 | 436718 | rhocb |  |  | 948 | 2.171 | 0.0862 | No |
| 11 | 556277 | cblb |  |  | 1038 | 2.056 | 0.0915 | No |
| 12 | 245702 | igf1rb |  |  | 1196 | 1.904 | 0.0941 | No |
| 13 | 565743\_1 | pld2 |  |  | 1216 | 1.885 | 0.1010 | No |
| 14 | 393881 | vps26a |  |  | 1281 | 1.824 | 0.1062 | No |
| 15 | 474327 | capza1b |  |  | 1384 | 1.736 | 0.1099 | No |
| 16 | 393586 | hsc70 |  |  | 1533 | 1.636 | 0.1117 | No |
| 17 | 100073336 | zgc:165502 |  |  | 1573 | 1.608 | 0.1169 | No |
| 18 | 561771 | acap1 |  |  | 1659 | 1.563 | 0.1204 | No |
| 19 | 100148154\_1 | agap1 |  |  | 1718 | 1.525 | 0.1246 | No |
| 20 | 796604 | il2rga |  |  | 1892 | 1.416 | 0.1248 | No |
| 21 | 562949 | ehd3 |  |  | 1983 | 1.373 | 0.1274 | No |
| 22 | 572485 | si:dkey-71h2.2 |  |  | 2060 | 1.344 | 0.1303 | No |
| 23 | 100147988 | chmp6a |  |  | 2217 | 1.281 | 0.1305 | No |
| 24 | 568170\_1 | eps15l1a |  |  | 2391 | 1.212 | 0.1298 | No |
| 25 | 565563 | kif5c |  |  | 2416 | 1.202 | 0.1338 | No |
| 26 | 100307098 | dnm1a |  |  | 2483 | 1.179 | 0.1364 | No |
| 27 | 100151129\_1 | rab11fip4b |  |  | 2658 | 1.124 | 0.1353 | No |
| 28 | 100001531 | rab22a |  |  | 2672 | 1.120 | 0.1394 | No |
| 29 | 572492 | pld1b |  |  | 2854 | 1.069 | 0.1379 | No |
| 30 | 550265 | zgc:110286 |  |  | 2858 | 1.069 | 0.1421 | No |
| 31 | 767667 | mvb12ba |  |  | 2871 | 1.066 | 0.1459 | No |
| 32 | 796345\_2 | dnajc6 |  |  | 2900 | 1.058 | 0.1492 | No |
| 33 | 550402 | arf4b |  |  | 2904 | 1.057 | 0.1533 | No |
| 34 | 562935 | si:dkey-4p15.3 |  |  | 2952 | 1.049 | 0.1560 | No |
| 35 | 100009637\_2 | wasla |  |  | 3137 | 1.004 | 0.1542 | No |
| 36 | 445196 | arf4a |  |  | 3169 | 0.999 | 0.1572 | No |
| 37 | 100334106 | iqsec1a |  |  | 3232 | 0.986 | 0.1591 | No |
| 38 | 406820 | actr2a |  |  | 3314 | 0.969 | 0.1604 | No |
| 39 | 563561 | LOC563561 |  |  | 3620 | 0.907 | 0.1543 | No |
| 40 | 570939 | LOC570939 |  |  | 3723 | 0.885 | 0.1546 | No |
| 41 | 571897 | rab8a |  |  | 3734 | 0.882 | 0.1578 | No |
| 42 | 569532\_1 | chmp2ba |  |  | 3911 | 0.846 | 0.1556 | No |
| 43 | 323579 | cltca |  |  | 4043 | 0.820 | 0.1547 | No |
| 44 | 100330183 | cltcl1 |  |  | 4192 | 0.795 | 0.1531 | No |
| 45 | 100151129\_2 | rab11fip4b |  |  | 4197 | 0.793 | 0.1562 | No |
| 46 | 556277\_1 | cblb |  |  | 4343 | 0.770 | 0.1546 | No |
| 47 | 793882 | git2a |  |  | 4352 | 0.769 | 0.1574 | No |
| 48 | 556132 | smap2 |  |  | 4604 | 0.734 | 0.1524 | No |
| 49 | 30637\_1 | mdm2 |  |  | 4722 | 0.715 | 0.1515 | No |
| 50 | 100331274 | itcha |  |  | 4738 | 0.713 | 0.1539 | No |
| 51 | 100148085 | ap2s1 |  |  | 4795 | 0.706 | 0.1549 | No |
| 52 | 100334729 | rab11fip2 |  |  | 4823 | 0.702 | 0.1568 | No |
| 53 | 751630 | zgc:153713 |  |  | 4826 | 0.702 | 0.1596 | No |
| 54 | 58129\_1 | fgfr3 |  |  | 4965 | 0.686 | 0.1579 | No |
| 55 | 101884351 | LOC101884351 |  |  | 5336 | 0.644 | 0.1487 | No |
| 56 | 100124652 | pard6b |  |  | 5488 | 0.635 | 0.1464 | No |
| 57 | 447839\_2 | ehd1b |  |  | 5626 | 0.623 | 0.1446 | No |
| 58 | 557199 | psd2 |  |  | 5728 | 0.614 | 0.1438 | No |
| 59 | 793623 | sh3kbp1 |  |  | 5815 | 0.606 | 0.1435 | No |
| 60 | 100005008 | LOC100005008 |  |  | 6110 | 0.576 | 0.1364 | No |
| 61 | 553266 | arrb1 |  |  | 6277 | 0.559 | 0.1334 | No |
| 62 | 58092\_1 | smad3a |  |  | 6307 | 0.555 | 0.1347 | No |
| 63 | 494477 | tfr1a |  |  | 6320 | 0.555 | 0.1365 | No |
| 64 | 569546 | ehd4 |  |  | 6407 | 0.546 | 0.1360 | No |
| 65 | 573631 | rab11fip3 |  |  | 6523 | 0.535 | 0.1344 | No |
| 66 | 566797 | zgc:114173 |  |  | 6585 | 0.529 | 0.1346 | No |
| 67 | novel.261 | - |  |  | 6610 | 0.527 | 0.1359 | No |
| 68 | 110437952 | LOC110437952 |  |  | 6631 | 0.525 | 0.1374 | No |
| 69 | 405821 | rab5b |  |  | 6719 | 0.518 | 0.1367 | No |
| 70 | 325084 | src |  |  | 6875 | 0.502 | 0.1338 | No |
| 71 | 797422 | stambpa |  |  | 6935 | 0.497 | 0.1339 | No |
| 72 | 798846 | si:ch211-199o1.2 |  |  | 7036 | 0.489 | 0.1326 | No |
| 73 | 494478 | tfr1b |  |  | 7066 | 0.486 | 0.1336 | No |
| 74 | 571453 | epn3b |  |  | 7095 | 0.483 | 0.1347 | No |
| 75 | 114447 | cxcr4b |  |  | 7160 | 0.476 | 0.1345 | No |
| 76 | 568737 | snx2 |  |  | 7241 | 0.470 | 0.1339 | No |
| 77 | 386807 | rab5c |  |  | 7243 | 0.470 | 0.1357 | No |
| 78 | 80959 | pard6gb |  |  | 7495 | 0.454 | 0.1295 | No |
| 79 | 368364 | sb:cb649 |  |  | 7502 | 0.454 | 0.1312 | No |
| 80 | 108191324 | LOC108191324 |  |  | 7553 | 0.451 | 0.1314 | No |
| 81 | 337386 | snx1a |  |  | 7740 | 0.440 | 0.1272 | No |
| 82 | 108191324\_1 | LOC108191324 |  |  | 7769 | 0.439 | 0.1281 | No |
| 83 | 100170829 | asap1a |  |  | 7886 | 0.432 | 0.1261 | No |
| 84 | 100333040 | si:ch1073-165f9.2 |  |  | 7925 | 0.429 | 0.1266 | No |
| 85 | 797187 | iqsec2b |  |  | 8003 | 0.423 | 0.1258 | No |
| 86 | 402889 | arfgap3 |  |  | 8016 | 0.423 | 0.1271 | No |
| 87 | 393902\_2 | rab7 |  |  | 8135 | 0.416 | 0.1250 | No |
| 88 | 100005341 | wipf1a |  |  | 8144 | 0.416 | 0.1264 | No |
| 89 | 108190803 | LOC108190803 |  |  | 8194 | 0.415 | 0.1265 | No |
| 90 | 492549 | pip5k1ab |  |  | 8298 | 0.413 | 0.1249 | No |
| 91 | 797355\_1 | iqsec3a |  |  | 8398 | 0.411 | 0.1234 | No |
| 92 | 794689 | zfyve16 |  |  | 8515 | 0.402 | 0.1213 | No |
| 93 | 336504 | arpc5a |  |  | 8597 | 0.395 | 0.1203 | No |
| 94 | 245701\_1 | igf1ra |  |  | 8653 | 0.391 | 0.1201 | No |
| 95 | 103909878 | LOC103909878 |  |  | 8683 | 0.389 | 0.1207 | No |
| 96 | 415179 | tsg101a |  |  | 8716 | 0.387 | 0.1213 | No |
| 97 | 322991 | acap3a |  |  | 8824 | 0.379 | 0.1194 | No |
| 98 | 100005033 | si:ch211-243a15.1 |  |  | 8828 | 0.379 | 0.1208 | No |
| 99 | 559329 | psd3l |  |  | 8994 | 0.368 | 0.1170 | No |
| 100 | novel.763 | - |  |  | 9036 | 0.365 | 0.1172 | No |
| 101 | 404040 | dab2 |  |  | 9070 | 0.362 | 0.1175 | No |
| 102 | 100330077 | rufy1 |  |  | 9085 | 0.361 | 0.1185 | No |
| 103 | 565859 | asap1b |  |  | 9091 | 0.361 | 0.1198 | No |
| 104 | 567272 | fam21c |  |  | 9231 | 0.351 | 0.1168 | No |
| 105 | 321695 | smurf1 |  |  | 9244 | 0.350 | 0.1178 | No |
| 106 | novel.57 | - |  |  | 9327 | 0.345 | 0.1166 | No |
| 107 | 100005449 | zfyve9a |  |  | 9485 | 0.335 | 0.1129 | No |
| 108 | 567272\_1 | fam21c |  |  | 9536 | 0.331 | 0.1127 | No |
| 109 | 415255 | wipf2b |  |  | 9561 | 0.329 | 0.1132 | No |
| 110 | 560461 | LOC560461 |  |  | 9628 | 0.325 | 0.1124 | No |
| 111 | 406414 | vta1 |  |  | 9933 | 0.308 | 0.1040 | No |
| 112 | 492364 | cbl |  |  | 9938 | 0.307 | 0.1051 | No |
| 113 | 101882851 | wu:fj66e11 |  |  | 10121 | 0.295 | 0.1005 | No |
| 114 | 100148736 | iqsec1b |  |  | 10237 | 0.289 | 0.0979 | No |
| 115 | 566849 | cyth4a |  |  | 10239 | 0.289 | 0.0991 | No |
| 116 | 394034 | waslb |  |  | 10311 | 0.284 | 0.0979 | No |
| 117 | 100329953 | arfgef1 |  |  | 10376 | 0.280 | 0.0970 | No |
| 118 | 386856 | pdgfra |  |  | 10436 | 0.277 | 0.0962 | No |
| 119 | 100136849 | zgc:172270 |  |  | 10500 | 0.273 | 0.0953 | No |
| 120 | 100005765 | spg20a |  |  | 10622 | 0.265 | 0.0925 | No |
| 121 | 555174 | wash1 |  |  | 10639 | 0.264 | 0.0931 | No |
| 122 | 794862\_1 | epn3a |  |  | 10734 | 0.259 | 0.0911 | No |
| 123 | 564814 | si:ch211-168b3.1 |  |  | 10787 | 0.256 | 0.0905 | No |
| 124 | 393550 | arpc5la |  |  | 10926 | 0.248 | 0.0871 | No |
| 125 | 570918 | si:ch211-150i13.1 |  |  | 10944 | 0.247 | 0.0875 | No |
| 126 | 393150 | sh3gl3b |  |  | 11077 | 0.240 | 0.0843 | No |
| 127 | 245701 | igf1ra |  |  | 11103 | 0.239 | 0.0845 | No |
| 128 | 793920 | il2rb |  |  | 11196 | 0.233 | 0.0825 | No |
| 129 | 492487\_1 | rab11a |  |  | 11286 | 0.228 | 0.0805 | No |
| 130 | 560578 | tgfbr1a |  |  | 11347 | 0.225 | 0.0795 | No |
| 131 | 321460 | ist1 |  |  | 11527 | 0.215 | 0.0747 | No |
| 132 | 100537448 | cyth3b |  |  | 11563 | 0.213 | 0.0744 | No |
| 133 | 114428 | arf1 |  |  | 11576 | 0.212 | 0.0749 | No |
| 134 | 561771\_1 | acap1 |  |  | 11614 | 0.210 | 0.0746 | No |
| 135 | 492695 | eea1 |  |  | 11713 | 0.205 | 0.0723 | No |
| 136 | 556641 | pld1a |  |  | 11723 | 0.205 | 0.0728 | No |
| 137 | 100004988 | si:ch211-207g17.2 |  |  | 11725 | 0.205 | 0.0736 | No |
| 138 | 326283 | smad3b |  |  | 11759 | 0.203 | 0.0733 | No |
| 139 | 334632 | ap2b1 |  |  | 11842 | 0.198 | 0.0715 | No |
| 140 | 378838\_1 | sh3gl1b |  |  | 11844 | 0.198 | 0.0723 | No |
| 141 | 568935 | git1 |  |  | 11911 | 0.195 | 0.0710 | No |
| 142 | 569403 | adrbk2 |  |  | 11980 | 0.192 | 0.0696 | No |
| 143 | 556104 | arpc5lb |  |  | 11981 | 0.192 | 0.0703 | No |
| 144 | 797181 | LOC797181 |  |  | 12052 | 0.188 | 0.0688 | No |
| 145 | 324332 | agap3 |  |  | 12219 | 0.178 | 0.0643 | No |
| 146 | 431725 | zgc:91909 |  |  | 12321 | 0.172 | 0.0618 | No |
| 147 | 619253 | snx1b |  |  | 12516 | 0.163 | 0.0562 | No |
| 148 | 494576\_1 | si:dkey-13a21.4 |  |  | 12522 | 0.163 | 0.0567 | No |
| 149 | 791453 | mhc1zba |  |  | 12541 | 0.162 | 0.0568 | No |
| 150 | 337737 | rab5aa |  |  | 12603 | 0.159 | 0.0555 | No |
| 151 | 561879 | unm\_sa1614 |  |  | 12642 | 0.157 | 0.0549 | No |
| 152 | 449831 | pip5k1ba |  |  | 12759 | 0.151 | 0.0518 | No |
| 153 | 558613 | chmp5a |  |  | 12765 | 0.151 | 0.0523 | No |
| 154 | 777731 | smap1 |  |  | 12918 | 0.145 | 0.0480 | No |
| 155 | 394099 | arrb2b |  |  | 13009 | 0.139 | 0.0457 | No |
| 156 | 798876 | pip5k1ca |  |  | 13026 | 0.139 | 0.0458 | No |
| 157 | 493612 | zgc:101810 |  |  | 13049 | 0.137 | 0.0456 | No |
| 158 | 140747 | cxcr4a |  |  | 13072 | 0.136 | 0.0455 | No |
| 159 | 550406 | grk1b |  |  | 13125 | 0.134 | 0.0443 | No |
| 160 | 100004330 | mvb12bb |  |  | 13174 | 0.131 | 0.0433 | No |
| 161 | 100005081 | si:ch211-207g17.3 |  |  | 13175 | 0.131 | 0.0439 | No |
| 162 | 336063 | arf6a |  |  | 13195 | 0.130 | 0.0438 | No |
| 163 | 503600 | cltcb |  |  | 13254 | 0.128 | 0.0424 | No |
| 164 | 560542 | epn2 |  |  | 13377 | 0.122 | 0.0391 | No |
| 165 | 567182 | snx6 |  |  | 13445 | 0.118 | 0.0374 | No |
| 166 | 393902\_1 | rab7 |  |  | 13530 | 0.114 | 0.0352 | No |
| 167 | 394243 | arpc1a |  |  | 13546 | 0.113 | 0.0352 | No |
| 168 | 571767 | si:ch211-135f11.1 |  |  | 13568 | 0.112 | 0.0349 | No |
| 169 | 445305\_2 | arpc4l |  |  | 13646 | 0.109 | 0.0329 | No |
| 170 | 406671 | epn1 |  |  | 13691 | 0.107 | 0.0320 | No |
| 171 | 406797 | snx5 |  |  | 13707 | 0.107 | 0.0319 | No |
| 172 | 335083 | capzb |  |  | 13723 | 0.106 | 0.0318 | No |
| 173 | 447863 | bin1b |  |  | 13830 | 0.100 | 0.0289 | No |
| 174 | 565743 | pld2 |  |  | 13902 | 0.096 | 0.0270 | No |
| 175 | 335474 | arpc1b |  |  | 13993 | 0.093 | 0.0245 | No |
| 176 | 445397 | chmp6b |  |  | 14023 | 0.091 | 0.0240 | No |
| 177 | 559634 | nedd4l |  |  | 14142 | 0.085 | 0.0205 | No |
| 178 | 103909750 | si:dkey-4m13.3 |  |  | 14303 | 0.078 | 0.0158 | No |
| 179 | 100170824 | pard6ga |  |  | 14339 | 0.077 | 0.0150 | No |
| 180 | novel.582 | - |  |  | 14385 | 0.075 | 0.0138 | No |
| 181 | 553374 | rab11fip1a |  |  | 14393 | 0.075 | 0.0139 | No |
| 182 | 393152 | strumpellin |  |  | 14440 | 0.073 | 0.0127 | No |
| 183 | 58129 | fgfr3 |  |  | 14448 | 0.072 | 0.0128 | No |
| 184 | 795505 | eps15 |  |  | 14579 | 0.066 | 0.0089 | No |
| 185 | 796345 | dnajc6 |  |  | 14634 | 0.063 | 0.0075 | No |
| 186 | 571626 | sh3gl3a |  |  | 14722 | 0.059 | 0.0049 | No |
| 187 | novel.922 | - |  |  | 14730 | 0.059 | 0.0050 | No |
| 188 | 565383 | cblc |  |  | 14820 | 0.055 | 0.0023 | No |
| 189 | 110438676 | LOC110438676 |  |  | 14839 | 0.054 | 0.0020 | No |
| 190 | 100329879 | cltb |  |  | 14942 | 0.049 | -0.0011 | No |
| 191 | 386967 | stam |  |  | 15098 | 0.041 | -0.0058 | No |
| 192 | 403050 | pard3ab |  |  | 15104 | 0.041 | -0.0058 | No |
| 193 | 777738 | vps37c |  |  | 15374 | 0.029 | -0.0142 | No |
| 194 | 352940 | fgfr2 |  |  | 15408 | 0.027 | -0.0152 | No |
| 195 | 565752 | si:dkey-23p11.2 |  |  | 15479 | 0.023 | -0.0173 | No |
| 196 | 415240 | cav2 |  |  | 15520 | 0.021 | -0.0185 | No |
| 197 | 415204 | arpc3 |  |  | 15534 | 0.020 | -0.0188 | No |
| 198 | 100332526 | LOC100332526 |  |  | 15612 | 0.017 | -0.0212 | No |
| 199 | 100333543 | dnm1b |  |  | 15632 | 0.016 | -0.0218 | No |
| 200 | 564152 | grk1a |  |  | 15661 | 0.014 | -0.0226 | No |
| 201 | 554861 | actr2b |  |  | 15691 | 0.012 | -0.0235 | No |
| 202 | 394001 | ap2m1b |  |  | 15702 | 0.011 | -0.0237 | No |
| 203 | 378838 | sh3gl1b |  |  | 15737 | 0.009 | -0.0248 | No |
| 204 | 566350 | gbf1 |  |  | 15759 | 0.008 | -0.0254 | No |
| 205 | 368822\_1 | arf3b |  |  | 15764 | 0.008 | -0.0255 | No |
| 206 | 406525 | dnm2b |  |  | 15794 | 0.007 | -0.0264 | No |
| 207 | 368323\_1 | si:ch211-204c21.1 |  |  | 15946 | 0.000 | -0.0312 | No |
| 208 | 794400 | sh3gl2b |  |  | 16035 | -0.003 | -0.0340 | No |
| 209 | 799274 | kif5ab |  |  | 16053 | -0.003 | -0.0345 | No |
| 210 | 794862 | epn3a |  |  | 16082 | -0.005 | -0.0354 | No |
| 211 | 557061 | igf2r |  |  | 16158 | -0.007 | -0.0377 | No |
| 212 | 570669 | asap2a |  |  | 16169 | -0.007 | -0.0380 | No |
| 213 | 393562 | vps28 |  |  | 16239 | -0.009 | -0.0402 | No |
| 214 | 559334 | dnm2a |  |  | 16269 | -0.010 | -0.0411 | No |
| 215 | 100330031 | itchb |  |  | 16323 | -0.012 | -0.0427 | No |
| 216 | 334108 | kif5bb |  |  | 16350 | -0.012 | -0.0435 | No |
| 217 | 796724 | cxcr2 |  |  | 16371 | -0.013 | -0.0441 | No |
| 218 | 494478\_1 | tfr1b |  |  | 16422 | -0.015 | -0.0456 | No |
| 219 | 378478 | egfra |  |  | 16429 | -0.015 | -0.0457 | No |
| 220 | novel.594 | - |  |  | 16482 | -0.017 | -0.0473 | No |
| 221 | 568064 | rabep1 |  |  | 16612 | -0.021 | -0.0513 | No |
| 222 | 337401 | ehd2b |  |  | 16647 | -0.022 | -0.0523 | No |
| 223 | 101884856\_1 | si:ch211-284f22.3 |  |  | 16936 | -0.032 | -0.0613 | No |
| 224 | 560581 | asap2b |  |  | 17013 | -0.034 | -0.0636 | No |
| 225 | 393880 | vps4b |  |  | 17091 | -0.036 | -0.0659 | No |
| 226 | 561659 | zfyve27 |  |  | 17179 | -0.040 | -0.0685 | No |
| 227 | 560446 | si:dkey-101k6.5 |  |  | 17193 | -0.040 | -0.0688 | No |
| 228 | 555340 | iqsec3b |  |  | 17242 | -0.043 | -0.0701 | No |
| 229 | 393351 | chmp5b |  |  | 17275 | -0.044 | -0.0710 | No |
| 230 | 334219\_1 | zgc:77650 |  |  | 17300 | -0.045 | -0.0716 | No |
| 231 | 100000160 | fgfr4 |  |  | 17448 | -0.051 | -0.0760 | No |
| 232 | 406318 | clta |  |  | 17484 | -0.053 | -0.0769 | No |
| 233 | 565434 | usp8 |  |  | 17506 | -0.054 | -0.0774 | No |
| 234 | 402861 | rab11fip1b |  |  | 17524 | -0.055 | -0.0777 | No |
| 235 | 553629 | pip5k1aa |  |  | 17647 | -0.060 | -0.0813 | No |
| 236 | 100009637 | wasla |  |  | 17655 | -0.061 | -0.0813 | No |
| 237 | 445154 | rab35b |  |  | 17663 | -0.061 | -0.0813 | No |
| 238 | 100009646 | acap3b |  |  | 17676 | -0.061 | -0.0814 | No |
| 239 | 767670 | vps4a |  |  | 17948 | -0.073 | -0.0898 | No |
| 240 | 103911953 | LOC103911953 |  |  | 18024 | -0.077 | -0.0918 | No |
| 241 | 558931 | arap3 |  |  | 18157 | -0.083 | -0.0957 | No |
| 242 | 100151491 | wipf1b |  |  | 18257 | -0.088 | -0.0985 | No |
| 243 | 406411 | rhoaa |  |  | 18330 | -0.091 | -0.1004 | No |
| 244 | 100001531\_1 | rab22a |  |  | 18350 | -0.092 | -0.1007 | No |
| 245 | 406669 | pdcd6ip |  |  | 18443 | -0.096 | -0.1032 | No |
| 246 | 399477 | arf2a |  |  | 18665 | -0.107 | -0.1098 | No |
| 247 | 567452 | grk5l |  |  | 18735 | -0.110 | -0.1116 | No |
| 248 | 101884856 | si:ch211-284f22.3 |  |  | 18784 | -0.112 | -0.1126 | No |
| 249 | 30739 | tgfbr2b |  |  | 18789 | -0.113 | -0.1123 | No |
| 250 | 558153 | ap2a1 |  |  | 18843 | -0.116 | -0.1135 | No |
| 251 | 393535 | chmp1a |  |  | 18849 | -0.116 | -0.1132 | No |
| 252 | 117507 | prkci |  |  | 18879 | -0.117 | -0.1137 | No |
| 253 | 100004931 | zgc:162872 |  |  | 18967 | -0.120 | -0.1160 | No |
| 254 | 641490 | arfgap2 |  |  | 18992 | -0.121 | -0.1163 | No |
| 255 | 562719 | rab11fip5a |  |  | 19011 | -0.122 | -0.1163 | No |
| 256 | 567055 | ehd2a |  |  | 19012 | -0.122 | -0.1159 | No |
| 257 | 406766 | chmp4bb |  |  | 19021 | -0.122 | -0.1156 | No |
| 258 | 393804 | amph |  |  | 19047 | -0.124 | -0.1159 | No |
| 259 | 335810 | vps36 |  |  | 19065 | -0.125 | -0.1160 | No |
| 260 | 100334572 | arfgef2 |  |  | 19124 | -0.128 | -0.1173 | No |
| 261 | 569358 | si:dkey-283b15.4 |  |  | 19157 | -0.129 | -0.1178 | No |
| 262 | 641572 | stambpb |  |  | 19281 | -0.135 | -0.1212 | No |
| 263 | 323203 | snx4 |  |  | 19288 | -0.135 | -0.1208 | No |
| 264 | 393164 | chmp4ba |  |  | 19448 | -0.142 | -0.1253 | No |
| 265 | 321378 | arrb2a |  |  | 19599 | -0.148 | -0.1295 | No |
| 266 | 566120 | grk7a |  |  | 19699 | -0.153 | -0.1320 | No |
| 267 | 406798 | rab11ba |  |  | 19700 | -0.153 | -0.1314 | No |
| 268 | 378724\_1 | arf5 |  |  | 19723 | -0.154 | -0.1315 | No |
| 269 | 393945 | rab5ab |  |  | 19731 | -0.154 | -0.1311 | No |
| 270 | 30639 | smad2 |  |  | 19779 | -0.156 | -0.1320 | No |
| 271 | 100144554 | il2rgb |  |  | 19913 | -0.162 | -0.1356 | No |
| 272 | 573437 | vps29 |  |  | 19924 | -0.163 | -0.1352 | No |
| 273 | 394094 | sh3glb2b |  |  | 20095 | -0.170 | -0.1400 | No |
| 274 | 245701\_2 | igf1ra |  |  | 20190 | -0.174 | -0.1423 | No |
| 275 | 100003902 | si:dkey-16l2.16 |  |  | 20237 | -0.176 | -0.1430 | No |
| 276 | 336839 | cdc42 |  |  | 20515 | -0.189 | -0.1511 | No |
| 277 | 334219 | zgc:77650 |  |  | 20519 | -0.189 | -0.1504 | No |
| 278 | 403031 | rab4a |  |  | 20603 | -0.194 | -0.1523 | No |
| 279 | 494576 | si:dkey-13a21.4 |  |  | 20660 | -0.197 | -0.1533 | No |
| 280 | 100333057 | tsg101b |  |  | 20662 | -0.197 | -0.1525 | No |
| 281 | 792928 | tgfbr1b |  |  | 20664 | -0.197 | -0.1518 | No |
| 282 | 792915 | zgc:194578 |  |  | 20743 | -0.200 | -0.1534 | No |
| 283 | 327029 | capza1a |  |  | 20750 | -0.201 | -0.1528 | No |
| 284 | 321051 | ap2m1a |  |  | 20766 | -0.202 | -0.1525 | No |
| 285 | 406621 | chmp3 |  |  | 20867 | -0.208 | -0.1549 | No |
| 286 | 560806 | grk5 |  |  | 20993 | -0.215 | -0.1580 | No |
| 287 | 436806 | rab11fip4a |  |  | 20998 | -0.215 | -0.1572 | No |
| 288 | 100009637\_1 | wasla |  |  | 21002 | -0.216 | -0.1565 | No |
| 289 | 378724 | arf5 |  |  | 21032 | -0.217 | -0.1565 | No |
| 290 | 447840 | pip5k1bb |  |  | 21133 | -0.223 | -0.1588 | No |
| 291 | 560761 | grk4 |  |  | 21154 | -0.224 | -0.1586 | No |
| 292 | 394091 | sh3gl2a |  |  | 21257 | -0.228 | -0.1609 | No |
| 293 | 100004625 | ccr11.1 |  |  | 21302 | -0.230 | -0.1614 | No |
| 294 | 327026 | arf2b |  |  | 21311 | -0.231 | -0.1607 | No |
| 295 | 325586\_1 | cyth1b |  |  | 21476 | -0.240 | -0.1650 | No |
| 296 | 569240 | rbsn |  |  | 21526 | -0.242 | -0.1656 | No |
| 297 | 100136849\_1 | zgc:172270 |  |  | 21633 | -0.248 | -0.1680 | No |
| 298 | 399482 | arpc5b |  |  | 21672 | -0.250 | -0.1682 | No |
| 299 | 474327\_2 | capza1b |  |  | 21678 | -0.250 | -0.1673 | No |
| 300 | 323695 | cav1 |  |  | 21810 | -0.257 | -0.1705 | No |
| 301 | 557582 | si:dkey-28n18.9 |  |  | 21857 | -0.260 | -0.1709 | No |
| 302 | novel.836 | - |  |  | 21900 | -0.262 | -0.1712 | No |
| 303 | 405821\_1 | rab5b |  |  | 21939 | -0.264 | -0.1713 | No |
| 304 | 405840 | chmp2bb |  |  | 22045 | -0.270 | -0.1736 | No |
| 305 | 101882925 | pard3aa |  |  | 22073 | -0.271 | -0.1734 | No |
| 306 | 554561 | traf6 |  |  | 22116 | -0.274 | -0.1736 | No |
| 307 | 386994 | rab11al |  |  | 22121 | -0.274 | -0.1727 | No |
| 308 | 245701\_3 | igf1ra |  |  | 22143 | -0.275 | -0.1722 | No |
| 309 | 561653 | vps45 |  |  | 22221 | -0.279 | -0.1736 | No |
| 310 | 100038776 | actr3b |  |  | 22285 | -0.283 | -0.1744 | No |
| 311 | 402885 | sh3glb2a |  |  | 22307 | -0.284 | -0.1740 | No |
| 312 | 406704 | spg21 |  |  | 22319 | -0.285 | -0.1732 | No |
| 313 | 58092 | smad3a |  |  | 22399 | -0.290 | -0.1746 | No |
| 314 | 664764 | asap3 |  |  | 22404 | -0.290 | -0.1735 | No |
| 315 | 561697 | vps35 |  |  | 22518 | -0.297 | -0.1759 | No |
| 316 | 569096 | rufy2 |  |  | 22578 | -0.300 | -0.1766 | No |
| 317 | 559690 | zmp:0000000660 |  |  | 22618 | -0.303 | -0.1767 | No |
| 318 | 406250 | actr3 |  |  | 22666 | -0.305 | -0.1769 | No |
| 319 | 449679 | cav3 |  |  | 22714 | -0.308 | -0.1772 | No |
| 320 | 567190 | git2b |  |  | 22777 | -0.312 | -0.1779 | No |
| 321 | 563459 | kif5ba |  |  | 22998 | -0.327 | -0.1836 | No |
| 322 | 406659 | rhoac |  |  | 23154 | -0.338 | -0.1872 | No |
| 323 | 568064\_1 | rabep1 |  |  | 23320 | -0.349 | -0.1910 | No |
| 324 | 373871 | grk7b |  |  | 23380 | -0.353 | -0.1915 | No |
| 325 | 799490 | LOC799490 |  |  | 23402 | -0.354 | -0.1908 | No |
| 326 | 492771 | snf8 |  |  | 23438 | -0.357 | -0.1905 | No |
| 327 | 566086 | kif5aa |  |  | 23616 | -0.368 | -0.1946 | No |
| 328 | 368278 | ldlrap1a |  |  | 23681 | -0.372 | -0.1952 | No |
| 329 | 563976 | wipf2a |  |  | 23775 | -0.379 | -0.1966 | No |
| 330 | novel.812 | - |  |  | 23795 | -0.380 | -0.1957 | No |
| 331 | 394098 | snx12 |  |  | 23850 | -0.383 | -0.1959 | No |
| 332 | 555737\_1 | prkcz |  |  | 23911 | -0.387 | -0.1963 | No |
| 333 | 559483\_1 | vps37b |  |  | 24084 | -0.398 | -0.2002 | No |
| 334 | 100003206 | arf6b |  |  | 24135 | -0.402 | -0.2001 | No |
| 335 | 793623\_1 | sh3kbp1 |  |  | 24430 | -0.416 | -0.2078 | No |
| 336 | 492497 | stam2 |  |  | 24447 | -0.417 | -0.2067 | No |
| 337 | 563511 | snx3 |  |  | 24469 | -0.418 | -0.2057 | No |
| 338 | 393754 | chmp7 |  |  | 24474 | -0.418 | -0.2041 | No |
| 339 | 100006041 | rhoab |  |  | 24773 | -0.436 | -0.2119 | No |
| 340 | 30671 | hsp70.3 |  |  | 24984 | -0.444 | -0.2168 | No |
| 341 | 798901 | bc2 |  |  | 25024 | -0.448 | -0.2162 | No |
| 342 | 327068 | arpc2 |  |  | 25071 | -0.452 | -0.2159 | No |
| 343 | 573376\_1 | hspa8 |  |  | 25212 | -0.461 | -0.2185 | No |
| 344 | 393456 | mvb12a |  |  | 25258 | -0.464 | -0.2181 | No |
| 345 | 568800 | rab31 |  |  | 25305 | -0.467 | -0.2177 | No |
| 346 | 436688 | vps26bl |  |  | 25314 | -0.468 | -0.2161 | No |
| 347 | 553764 | zgc:109949 |  |  | 25328 | -0.469 | -0.2146 | No |
| 348 | 555737 | prkcz |  |  | 25364 | -0.472 | -0.2139 | No |
| 349 | 563633 | smurf2 |  |  | 25533 | -0.487 | -0.2173 | No |
| 350 | 570918\_1 | si:ch211-150i13.1 |  |  | 25662 | -0.500 | -0.2194 | No |
| 351 | 100126123 | hsp70.1 |  |  | 25714 | -0.503 | -0.2190 | No |
| 352 | 100329948 | eps15l1b |  |  | 25916 | -0.521 | -0.2233 | No |
| 353 | 100149572 | arfgap1 |  |  | 26260 | -0.553 | -0.2320 | No |
| 354 | 368323 | si:ch211-204c21.1 |  |  | 26383 | -0.565 | -0.2336 | No |
| 355 | 334911 | sh3glb1b |  |  | 26447 | -0.572 | -0.2333 | No |
| 356 | 550286 | hrasa |  |  | 26472 | -0.575 | -0.2318 | No |
| 357 | 100151196 | LOC100151196 |  |  | 26551 | -0.584 | -0.2320 | No |
| 358 | 791153 | ldlrap1b |  |  | 26552 | -0.584 | -0.2296 | No |
| 359 | 796604\_1 | il2rga |  |  | 26771 | -0.606 | -0.2342 | No |
| 360 | 30637 | mdm2 |  |  | 26774 | -0.606 | -0.2318 | No |
| 361 | 415190 | zgc:86896 |  |  | 26776 | -0.607 | -0.2294 | No |
| 362 | 100002190 | cyth3a |  |  | 26874 | -0.617 | -0.2301 | No |
| 363 | 100149843 | pip5kl1 |  |  | 27094 | -0.639 | -0.2345 | No |
| 364 | 103909792 | LOC103909792 |  |  | 27107 | -0.640 | -0.2323 | No |
| 365 | 101882992 | zfyve9b |  |  | 27150 | -0.643 | -0.2311 | No |
| 366 | 110439374 | LOC110439374 |  |  | 27394 | -0.657 | -0.2362 | No |
| 367 | 565109 | agap2 |  |  | 27527 | -0.676 | -0.2377 | No |
| 368 | 553656 | hrasb |  |  | 27554 | -0.680 | -0.2358 | No |
| 369 | 321938 | cyth1a |  |  | 27695 | -0.700 | -0.2375 | No |
| 370 | 100005753 | sh3gl1a |  |  | 27971 | -0.738 | -0.2433 | Yes |
| 371 | 796454 | rab35a |  |  | 28064 | -0.751 | -0.2432 | Yes |
| 372 | 445047 | arf3a |  |  | 28133 | -0.761 | -0.2423 | Yes |
| 373 | 110438369 | LOC110438369 |  |  | 28160 | -0.766 | -0.2401 | Yes |
| 374 | 556226 | cdc42l2 |  |  | 28186 | -0.770 | -0.2378 | Yes |
| 375 | 436828 | rab11bb |  |  | 28230 | -0.776 | -0.2361 | Yes |
| 376 | 100148154 | agap1 |  |  | 28250 | -0.778 | -0.2336 | Yes |
| 377 | 100141338 | cyth4b |  |  | 28259 | -0.779 | -0.2308 | Yes |
| 378 | 436839 | rab10 |  |  | 28337 | -0.793 | -0.2301 | Yes |
| 379 | 560461\_1 | LOC560461 |  |  | 28359 | -0.797 | -0.2276 | Yes |
| 380 | 797355 | iqsec3a |  |  | 28379 | -0.800 | -0.2250 | Yes |
| 381 | 569096\_1 | rufy2 |  |  | 28477 | -0.815 | -0.2248 | Yes |
| 382 | 567190\_1 | git2b |  |  | 28521 | -0.824 | -0.2229 | Yes |
| 383 | 323579\_1 | cltca |  |  | 28543 | -0.829 | -0.2203 | Yes |
| 384 | 791212 | zgc:158296 |  |  | 28684 | -0.854 | -0.2213 | Yes |
| 385 | 407684 | vps25 |  |  | 28879 | -0.894 | -0.2239 | Yes |
| 386 | 799278 | si:ch211-168k14.2 |  |  | 29073 | -0.936 | -0.2263 | Yes |
| 387 | 334219\_2 | zgc:77650 |  |  | 29103 | -0.943 | -0.2235 | Yes |
| 388 | 114456 | arpc4 |  |  | 29163 | -0.962 | -0.2215 | Yes |
| 389 | 325586 | cyth1b |  |  | 29278 | -0.995 | -0.2212 | Yes |
| 390 | 449549 | zgc:100918 |  |  | 29484 | -1.058 | -0.2235 | Yes |
| 391 | 445305\_1 | arpc4l |  |  | 29885 | -1.197 | -0.2315 | Yes |
| 392 | 561288 | grk6 |  |  | 30012 | -1.252 | -0.2305 | Yes |
| 393 | 393142 | ccdc53 |  |  | 30255 | -1.369 | -0.2327 | Yes |
| 394 | 556277\_2 | cblb |  |  | 30334 | -1.406 | -0.2296 | Yes |
| 395 | 492487 | rab11a |  |  | 30569 | -1.561 | -0.2308 | Yes |
| 396 | 336368\_1 | vps37a |  |  | 30591 | -1.578 | -0.2252 | Yes |
| 397 | 334175 | hgs |  |  | 30624 | -1.608 | -0.2198 | Yes |
| 398 | 405810 | ehd1a |  |  | 30646 | -1.632 | -0.2140 | Yes |
| 399 | 387529 | ldlra |  |  | 30808 | -1.767 | -0.2121 | Yes |
| 400 | 100151129 | rab11fip4b |  |  | 30821 | -1.778 | -0.2054 | Yes |
| 401 | 393881\_1 | vps26a |  |  | 31049 | -2.026 | -0.2045 | Yes |
| 402 | 406684 | vps26b |  |  | 31166 | -2.178 | -0.1996 | Yes |
| 403 | 245702\_1 | igf1rb |  |  | 31247 | -2.324 | -0.1928 | Yes |
| 404 | novel.946 | - |  |  | 31344 | -2.513 | -0.1859 | Yes |
| 405 | 560210 | hsp70l |  |  | 31396 | -2.640 | -0.1770 | Yes |
| 406 | 386856\_1 | pdgfra |  |  | 31415 | -2.695 | -0.1668 | Yes |
| 407 | 571431 | LOC571431 |  |  | 31422 | -2.716 | -0.1562 | Yes |
| 408 | 568170\_2 | eps15l1a |  |  | 31526 | -2.993 | -0.1476 | Yes |
| 409 | 100003902\_1 | si:dkey-16l2.16 |  |  | 31547 | -3.046 | -0.1361 | Yes |
| 410 | 792828 | pard6a |  |  | 31747 | -4.071 | -0.1262 | Yes |
| 411 | 436718\_1 | rhocb |  |  | 31770 | -4.295 | -0.1098 | Yes |
| 412 | 793158 | cdc42l |  |  | 31794 | -4.556 | -0.0924 | Yes |
| 413 | 474327\_1 | capza1b |  |  | 31799 | -4.600 | -0.0742 | Yes |
| 414 | 336426\_1 | chmp1b |  |  | 31821 | -4.782 | -0.0559 | Yes |
| 415 | 550542\_1 | chmp4c |  |  | 31822 | -4.783 | -0.0368 | Yes |
| 416 | 792229 | sh3glb1a |  |  | 31834 | -4.854 | -0.0179 | Yes |
| 417 | 406837 | rnf41 |  |  | 31865 | -4.931 | 0.0008 | Yes |
Table: GSEA details [plain text format]

  

Fig 2: ENDOCYTOSIS(DRE04144)      
 Blue-Pink O' Gram in the Space of the Analyzed GeneSet

  

Fig 3: ENDOCYTOSIS(DRE04144): Random ES distribution      
 Gene set null distribution of ES for **ENDOCYTOSIS(DRE04144)**
